# Weight Change and Risk of Venous Thromboembolism: The Tromsø Study

**DOI:** 10.1371/journal.pone.0168878

**Published:** 2016-12-20

**Authors:** Lars Daae Horvei, Sigrid K. Brækkan, John-Bjarne Hansen

**Affiliations:** 1 K.G. Jebsen Thrombosis Research and Expertise Center, Department of Clinical Medicine, UiT The Arctic University of Norway, Tromsø, Norway; 2 Division of Internal Medicine, University Hospital of North Norway, Tromsø, Norway; Maastricht University Medical Center, NETHERLANDS

## Abstract

**Background:**

Obesity is a major risk factor for venous thromboembolism (VTE), but it is unknown to what extent weight change over time affects VTE risk.

**Aims:**

To investigate the association between weight change and risk of incident VTE in a population-based cohort with repeated measurements.

**Methods:**

Participant data were collected from the Tromsø 3 (1986–87), 4 (1994–95), 5 (2000–01) and 6 (2007–08) surveys. Subjects who attended two subsequent or more surveys were included (n = 17802), and weight change between the surveys was calculated. Person-time at risk was accrued from the second of two subsequent vists until the next survey, the date of an incident VTE, migration, death or study end (December 31^st^ 2012), whichever came first. Cox regression models were used to calculate risk of VTE according to change in body weight.

**Results:**

There were 302 incident VTE events during a median of 6.0 years of follow-up. Subjects who gained most weight (7.5–40.0 kg weight gain) had a 1.9-fold higher risk of VTE compared to those with no or a moderate (0–7.4 kg) weight gain (HR 1.92; 95% CI 1.38–2.68). The VTE risk by ≥7.5 kgs over no or moderate (0–7.4 kg) weight gain was highest (HR 3.75; 95% 1.83–7.68) in subjects with baseline body mass index (BMI) ≥30 kg/m^2^. There was a joint effect of weight gain and baseline BMI on VTE risk. Those with BMI ≥30 who gained ≥7.5 kgs had a 6.6-fold increased risk (HR 6.64; 95% CI 3.61–12.22) compared to subjects with BMI <25 and no or moderate (0–7.4 kg) weight gain.

**Conclusions:**

Our findings imply that further weight gain is a considerable risk factor for VTE, particularly in obese individuals.

## Introduction

Overweight and obesity are major risk factors for venous thromboembolism (VTE). Results from prospective cohorts have consistently shown a 2 to 3-fold increased risk of VTE in obese subjects [1–7]. To date, the underlying mechanism for this association remains not fully understood [8]. Although obesity is a shared risk factor for arterial and venous thrombosis, the impact of body fat distribution and the cardiometabolic disturbances associated with central obesity, differs for the association between obesity and arterial and venous thrombosis [2]. Whereas cardiometabolic disturbances secondary to obesity are of major importance in the pathogenesis of myocardial infarction, other consequences of obesity, such as venous stasis, may be of more importance for VTE [9].

Modifiable risk factors, such as obesity, may lead to an underestimation of true associations between exposure and outcome in cohort studies. Analyses based on single [1–5] and repeated [6, 7] measures of obesity, however, have displayed essentially similar risk estimates for VTE. Although time-varying analyses based on repeated measures gives a more accurate estimate of the association between a modifiable risk factor and outcome, it does not assess the impact of intra-individual changes of a risk factor on the outcome. To our knowledge, no previous study have investigated whether changes in body weight are associated with risk of VTE. Considering the linear relationship between obesity measures and VTE risk [8], it is assumed that voluntary weight reduction in overweight persons will be beneficial, whereas involuntary weight loss (e.g. due to cancer or other diseases) may have an adverse effect on VTE risk. Conversely, weight gain may lead to increased risk of VTE. Actually, several studies have shown that weight gain, weight loss and/or fluctuations in body weight are all associated with future risk of arterial cardiovascular diseases as well as all-cause mortality [10–17]. Even within the “normal” weight range, weight gain is associated with higher risk of coronary heart disease [15].

In the present study, we aimed to investigate whether weight change was associated with VTE risk, and to elucidate whether weight change was a risk factor for VTE independent of obesity status. Therefore, we assessed the risk of VTE according to changes in body weight within a prospective population-based cohort with four repeated measurements 6–8 years apart, with and without adjustment for the attained body mass index.

## Materials and Methods

### Study population

As described previously [2], the Tromsø Study is a single-center, prospective, population-based health study, with repeated health surveys of inhabitants in Tromsø, Norway. The third survey was carried out in the period 1986–1987, the fourth in 1994–1995, the fifth in 2000–01 and the sixth in 2007–08. Members of the population living in the municipality of Tromsø, Norway, were invited to participate in these surveys. The overall participation rate was high, ranging from 66% to 78% in the different surveys. Subjects who attended two or more subsequent surveys were included in our study. A detailed description of study participation has been published previously [18]. Subjects with a history of VTE at baseline (*n* = 185) were excluded from the study. Thus, our study population consisted of 17 802 unique subjects, aged 25–89 years. The study was approved by the Regional Committee of Medical and Health Research Ethics, and all participants gave their informed written consent to participate.

### Measurements

Exposure information was collected by self-administered questionnaires, physical examination and non-fasting blood samples, as described in detail previously [2]. Body height and weight was measured by study personnel with subjects wearing light clothing and no shoes. Information on cancer before and during follow-up was obtained from the cancer registry of Norway. The cancer registry is considered complete and valid, and evaluations have reported 98.8% completeness, with 94% microscopically confirmed diagnoses [19].

### Outcome assessment of venous thromboembolism

All first lifetime VTE events during follow-up were recorded from the date of enrollment to the end of the study period. The VTE events were classified as unprovoked or provoked on the basis of provoking factors at the time of diagnosis. A detailed description of the outcome assessment has previously been published elsewhere [5]. In short, all incident VTEs in the region were identified using the hospital discharge diagnosis registry, the autopsy registry and the radiology procedure registry from University Hospital of North Norway (UNN), which is the sole provider of relevant diagnostic radiology in the area. All events were validated by trained personnel, requiring signs and symptoms of DVT or PE combined with objectiveconfirmation by radiological procedures, which resulted in treatment initiation unless contraindications were specified.

### Statistical analyses

**Fig 1:**
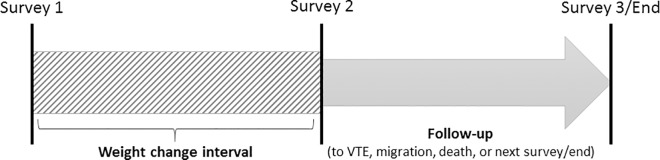
Overview of exposure and follow-up. Exposure was recorded as the change in body weight between two subsequent surveys. Subjects were followed until the date of a third survey or December 31, 2012. Subjects who attended three or more subsequent surveys thus contributed with multiple exposures and follow-up periods.

Statistical analyses were carried out using Stata version 14 (Statacorp LP). Weight change between two subsequent visits was calculated, and subjects were then followed from the date of the second visit until a VTE event, the next visit (or the median date of visit for the next survey if the survey was not attended), migration, death or study end (December 31^st^ 2012), whichever came first. Individuals who attended three or four subsequent visits contributed with multiple observation periods (Fig 1), yielding 27 376 observations in total. A detailed description of study participation and follow-up is presented in the supplementary material.

Time-varying cox-proportional hazards regression models were used to estimate hazard ratios (HR) with 95% confidence intervals (CI) for VTE across categories of change in body weight. Subjects with no or a moderate weight gain (0–7.5 kg) were used as reference, and were compared with subjects with any weight loss (-0.1 to -56.6 kg) and subjects with a weight gain ≥7.5 kg (the top 15 percent). We also performed analyses across quintiles of changes in body weight. Analyses were performed in three adjustments models, using age as time-scale. Model 1 was adjusted for sex, whereas model 2 included the attained BMI measured at the second visit. Other potential confounders such as smoking, systolic blood pressure, triglycerides, HDL-cholesterol and self-reported diabetes mellitus were not associated with VTE in the Tromsø Study [20, 21], and were therefore not included in the multivariable models. The proportional hazard assumption was verified by evaluating the parallelism between the curves of the log–log survivor function for different categories of the variables. Statistical interactions between weight change, BMI and the other variables in the models were tested, and there was a significant interaction between BMI and weight change. We therefore stratified the study population according to BMI status at the start of the weight change interval (<25 vs 25–29.9 and ≥30 kg/m^2^). We also performed analyses separately for provoked and unprovoked VTE. Because of limited statistical power, subjects with BMI ≥25 were merged into one strata in these analyses.

Cancer is a strong risk factor for VTE, and is often associated with involuntary weight loss. The risk of VTE is particularly high immediately before [22] and the first years after [23] cancer diagnosis. For sensitivity purposes, we therefore performed analyses where subjects with incident cancer were censored six months prior to the cancer diagnosis, but only if the time span between cancer and VTE diagnosis was less than five years. As weight variability also is related to mortality [12, 14], the observed associations could be influcenced by competing risk by death. We therefore applied The Fine–Gray model as a sensitivity analysis to account for death as a competing event [24].

## Results

**Fig 2:**
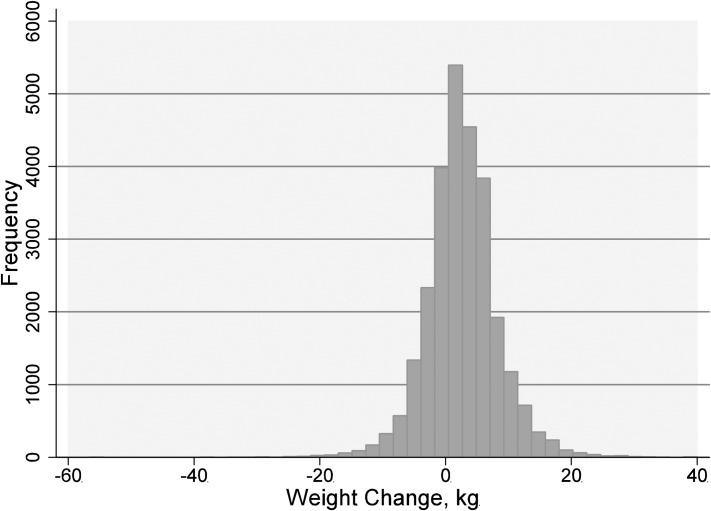
Distribution of weight change (in kgs) in the study population

The mean age at study entry was 54±14 years and 46.2% (n = 12,661) were men. The mean weight change in the study population was +2.6 kg, and the weight change in the cohort displayed a normal distribution (Fig 2).

Characteristics of subjects at study entry across weight change intervals are shown in Table 1. Subjects who experienced the most profound weight gain (7.5 kg or more) had the lowest mean age and the highest BMI at entry, while subjects in the middle interval (0–7.4 kg weight gain) were oldest and had the lowest BMI and percentage of smokers at study entry. The distribution of traditional cardiovascular risk factors at study entry were essentially similar across categories of weight change, except for a higher prevalence of diabetes among subjects who experienced weight loss. There were 302 incident VTEs during a median follow-up of 6.0 years, of which 120 (40%) were classified as unprovoked. The overall crude incidence rate was 1.8 (95% CI 1.6–2.1) per 1000 person-years.

**Table 1:** Baseline Characteristics of the Study Population Across Categories of Weight Change

	Category of Weight change		
	56.6–0.1 kg loss	0–7.4 kg gain	7.5–40.0 kg gain
n	8547	14331	4501
Men, %	48.3 (4124)	45.9 (6581)	43.5 (1958)
Age, years	59.0 ±14.3	52.9 ± 13.3	47.1 ±12.5
BMI, kg/m^2^	24.7 ±3.9	25.7 ±3.6	28.6 ±4.3
Body weight, kg	70.2 ± 13.2	73.8 ±12.8	83.3 ±14.1
Smoking, %	36.1 (3087)	31.6 (4530)	28.4 (1280)
Systolic blood pressure, mm Hg	139 ± 23	136 ±20	136 ±19
Total cholesterol, mmol/L	6.0 ±1.2	6.1 ±1.2	6.2 ±1.2
HDL cholesterol, mmol/L	1.6 ± 0.4	1.5 ±0.4	1.4 ±0.4
Triglycerides, mmol/L	1.4 ± 0.8	1.5 ±1.0	1.8 ±1.2
Diabetes mellitus, %	5.5 (468)	1.7 (238)	1.8 (81)

Values are means ±1 SD or percentage with numbers in brackets.

Risk estimates for VTE according to weight change are shown in Table 2. Subjects with the highest weight gain (≥7.5 kg) had a 1.9-fold increased risk of VTE (HR 1.93; 95% CI 1.38–2.68), and this risk was modestly attenuated after adjustment for BMI (HR 1.63, 95% CI 1.15–2.30 In stratified analyses, the risk of VTE according to weight change was most pronounced among obese subjects (baseline BMI ≥30 kg/m^2^). In obese subjects, weight gain ≥ 7.5 kg was associated with a 3.8-fold increased risk of VTE (HR 3.75; 95% CI 1.83–7.68). Overall, there was a trend towards a slightly increased VTE risk for subjects who lost weight (56.6–0.1 kg weight loss) (HR 1.15, 95% CI 0.89–1.49 in the model adjusted for BMI). The risk was most pronounced in overweight subjects (HR 1.43; 95% CI 0.95–2.13).

**Table 2:** Incidence Rates (IR) and Hazard Ratios (HR) with 95% Confidence Intervals (CI) for Venous Thromboembolism (VTE) According to Changes in Body Weight, Stratified by BMI at the Start of the Weight Change Interval

Body weight change	Person-years of follow-up	Events	IR*(95% CI)	Model 1^a^	Model 2^b^
				HR (95% CI)	HR (95% CI)
*All subjects*					
56.6–0.1 kg loss	49 050	120	2.45 (2.05–2.93)	1.06 (0.83–1.37)	1.15 (0.89–1.49)
0–7.4 kg gain	87 767	132	1.50 (1.27–1.78)	Ref	Ref
7.5–40.0 kg gain	28 022	50	1.78 (1.35–2.35)	1.93 (1.38–2.68)	1.63 (1.15–2.30)
*BMI <25*					
23.9–0.1 kg loss	24 846	30	1.21 (0.84–1.73)	0.74 (0.47–1.17)	0.78 (0.47–1.29)
0–7.4 kg gain	54 616	53	0.97 (0.74–1.27)	Ref	Ref
7.5–39.0 kg gain	16 815	19	1.13 (0.72–1.77)	1.86 (1.09–3.16)	1.74 (0.96–3.18)
*BMI 25–29*.*9*					
31.6–0.1 kg loss	18 028	65	3.61 (0.28–4.60)	1.24 (0.87–1.76)	1.43 (0.95–2.13)
0–7.4 kg gain	27 556	63	2.29 (1.79–2.93)	Ref	Ref
7.5–40.0 kg gain	8 639	15	1.74 (1.05–2.88)	1.21 (0.69–2.15)	1.00 (0.53–1.89)
*BMI ≥30*					
56.6–0.1 kg loss	6 176	25	4.05 (2.74–5.99)	1.24 (0.66–2.33)	1.01 (0.51–2.00)
0–7.4 kg gain	5 584	16	2.87 (1.76–4.68)	Ref	Ref
7.5–33.0 kg gain	2 567	16	6.23 (3.82–10.17)	3.75 (1.83–7.68)	4.70 (2.17–10.18)

*Crude incidence rates per 1000 person-years

^a^ Model 1: Age as time-scale, adjusted for sex

^b^ Model 2: Model 1 + adjusted for attained BMI

Corresponding analyses across quintiles of change in body weight (S1 Table) yielded similar results, with a somewhat stronger association for the quintile with the highest weight loss (HR 1.66; 95% CI 1.07–2.60.

**Fig 3:**
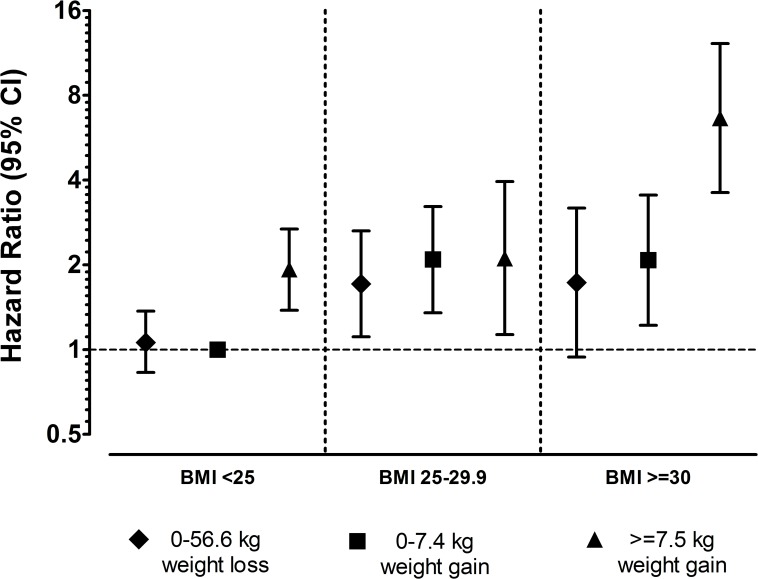
Hazard Ratios for Venous Thromboembolism According to Changes in Body Weight and Body Mass Index Measured at the Start of the Weight Change Interval. Subjects with BMI <25 kg/m^2^ and 0–7.4 kg weight gain were used as reference. Age as time-scale, adjusted for sex.

Hazard ratios for VTE according to weight change across BMI strata are presented in Fig 3. Compared to normal-weight subjects (baseline BMI <25 kg/m^2^) with no or moderate weight gain, obese subjects with the highest weight gain had a 6.6-fold increased risk of VTE (HR 6.64; 95% CI 3.61–12.22).

To investigate whether weight change had differential associations on risk of unprovoked and provoked VTE, we estimated hazard ratios for the two VTE types separately (Table 3). We found no significant associations between weight change and provoked VTE in any BMI strata. Weight gain (≥7.5 kg) was associated with a 2.6-fold increased risk of unprovoked VTE among normal-weight individuals (HR 2.57; 95% CI 1.23–5.37), and a 2.1-fold increased risk among overweight/obese (BMI ≥25 kg/m2) (HR 2.11; 95% CI 1.13–3.95). The risk estimates were not affected by adjustment for attained BMI.

**Table 3:** Crude Incidence Rates (IR) and Hazard Ratios (HR) with 95% Confidence Intervals (CI) for Provoked and Unprovoked Venous Thromboembolism (VTE) According to Changes in Body Weight. Stratified According to BMI at the start of the weight change interval.

Body weight change	Person-years of follow-up	Events		IR*	Model 1^a^	Model 2^b^
					HR (95% CI)	HR (95% CI)
**Provoked VTE**						
*BMI <25*						
23.6–0.1 kg loss	25 044		21	0.84 (0.55–1.29)	0.96 (0.54–1.70)	1.16 (0.61–2.21)
0–7.4 kg gain	54 813		30	0.55 (0.38–0.78)	Ref	Ref
7.5–30.2 kg gain	16 871		8	0.47 (0.24–0.95)	1.35 (0.61–2.96)	1.06 (0.44–2.55)
*BMI ≥25*						
56.6–0.1 kg loss	24 423		60	2.46 (1.91–3.16)	1.38 (0.94–2.03)	1.48 (1.00–2.21)
0–7.4 kg gain	33 260		47	1.41 (1.06–1.88)	Ref	Ref
7.5–40.0 kg gain	11 232		16	1.42 (0.87–2.33)	1.71 (0.96–3.04)	1.50 (0.82–2.73)
**Unprovoked VTE**						
*BMI <25*						
23.6–0.1 kg loss	25 044		9	0.42 (0.28–0.63)	0.47 (0.22–1.03)	0.41 (0.17–0.96)
0–7.4 kg gain	54 813		23	0.36 (0.19–0.69)	Ref	Ref
7.5–30.2 kg gain	16 871		11	0.65 (0.36–1.18)	2.57 (1.23–5.37)	3.07 (1.33–7.08)
*BMI ≥25*						
56.6–0.1 kg loss	24 423		30	0.96 (0.68–1.36)	1.00 (0.60–1.67)	0.97 (0.57–1.64)
0–7.4 kg gain	33 260		32	1.23 (0.86–1.76)	Ref	Ref
7.5–40.0 kg gain	11 232		15	1.34 (0.81–2.22)	2.11 (1.13–3.95)	2.15 (1.10–4.21)

*Crude incidence rates per 1000 person-years

^a^ Model 1: Age as time-scale, adjusted for sex

^b^ Model 2: Model 1 + adjusted for attained BMI

In order to investigate whether weight change attributed to cancer influenced the VTE risk, we repeated the analyses with incident cancer as a competing event (Table 4). In these analyses, the risk estimates for unprovoked VTE remained essentially unchanged, while the association between weight loss and provoked VTE among subjects with BMI >25 was more pronounced (HR adjusted for BMI 1.96, 95% CI 1.22–3.15). In contrast, the risk estimates for weight loss were slightly attenuated and no longer statistically significant when competing risk by death was taken into account (Table 4).

**Table 4:** Hazard Ratios (HR) with 95% Confidence Intervals (CI) for Provoked and Unprovoked Venous Thromboembolism According to Changes in Body Weight, Competing Risk by Cancer and Competing risk by Death Analysis

	Competing risk by cancer analysis				Competing risk by death analysis			
Body weight change	Person-years of follow-up	Events	Model 1^a^	Model 2^b^	Person-years of follow-up	Events	Model 1^a^	Model 2^b^
			HR (95% CI)	HR (95% CI)			SHR^c^ (95% CI)	SHR^c^ (95% CI)
**Provoked VTE**								
*BMI <25*								
23.6–0.1 kg loss	23 262	9	0.51 (0.23–1.13)	0.63 (0.26–1.50)	25 044	21	0.81 (0.46–1.42)	0.99 (0.52–1.90)
0–7.4 kg gain	52 428	23	Ref	Ref	54 813	30	Ref	Ref
7.5–30.2 kg gain	16 228	5	1.14 (0.43–3.05)	0.88 (0.29–2.65)	16 871	8	1.38 (0.63–3.03)	1.09 (0.46–2.57)
*BMI ≥25*								
56.6–0.1 kg loss	22 035	49	1.79 (1.13–2.83)	1.96 (1.22–3.15)	24 423	60	1.21 (0.82–1.78)	1.31 (0.88–1.93)
0–7.4 kg gain	30 742	30	Ref	Ref	33 260	47	Ref	Ref
7.5–40.0 kg gain	10 639	8	1.32 (0.60–2.90)	1.12 (0.49–2.53)	11 232	16	1.83 (1.03–3.26)	1.61 (0.90–2.88)
**Unprovoked VTE**								
*BMI <25*								
23.6–0.1 kg loss	23 262	8	0.49 (0.21–1.12)	0.44 (0.18–1.09)	25 044	9	0.42 (0.20–0.90)	0.36 (0.17–0.78)
0–7.4 kg gain	52 428	20	Ref	Ref	54 813	23	Ref	Ref
7.5–30.2 kg gain	16 228	9	2.41 (1.07–5.41)	2.75 (1.09–6.92)	16 871	11	2.59 (1.24–5.38)	3.04 (1.38–6.68)
*BMI ≥25*								
56.6–0.1 kg loss	22 035	27	0.98 (0.58–1.67)	0.96 (0.55–1.67)	24 423	30	0.88 (0.54–1.45)	0.85 (0.52–1.39)
0–7.4 kg gain	30 742	30	Ref	Ref	33 260	32	Ref	Ref
7.5–40.0 kg gain	10 639	15	2.22 (1.18–4.19)	2.22 (1.23–4.37)	11 232	15	2.34 (1.25–4.39)	2.40 (1.22–4.76)

^a^ Model 1: Age as time-scale, adjusted for sex

^b^ Model 2: Model 1 + adjusted for attained BMI

^**c**^ Subdistribution hazard ratio

## Discussion

In the present study, weight gain was associated with higher risk of VTE, which was mediated by an increased risk of unprovoked VTE events. The risk estimates were only modestly affected by adjustment for the attained BMI. Weight gain showed strongest association with VTE risk in already obese subjects, suggesting a joint effect of baseline BMI and weight gain. We also found an unexpected slightly increased risk of provoked VTE by weight loss in overweight and obese subjects. The association between weight loss and risk of provoked VTE was stronger when subjects who developed cancer were censored six monts prior to the cancer diagnosis, but was attenuated and no longer significant in competing risk by death analysis.

Weight gain is robustly associated with a variety of negative health outcomes, including cardiovascular disease [25], diabetes [10] and hypertension [11]. Accordingly, we found that weight gain was associated with increased risk of VTE, particularly among obese subjects. As there is a linear relationship between obesity and VTE risk, it is not surprising that increase in body weight increased the risk of VTE. The prothrombotic changes seen in individuals with obesity may contribute to the VTE risk by weight gain. Population-based studies have shown that chronic inflammation, assessed by CRP, is consistently associated with obesity [26, 27], and inflammation stimulates synthesis of plasminogen activator inhibitor 1 (PAI-1), tissue factor (TF), fibrinogen and potentially other factors involved in the coagulation cascade [28, 29]. Plasma levels of PAI-1 and factor VIII are elevated in obesity [30], and high levels of these factors have been associated with increased VTE risk in several studies [31, 32]. Recently, we have reported that chronic inflammation, assessed by repeated measures of CRP, was associated with increased VTE risk and partly explained the association between obesity and VTE risk, particularly in women [33]. Although excessive amounts of certain clotting factors and lack of inhibitors have been associated with increased risk of VTE [34], activation of coagulation with subsequent thrombus formation occurs only after initiation of the coagulation cascade in the presence of TF [35]. Adipokines are a group of hormone-like substances secreted from adipose tissue, which interact with the coagulation system in various ways [36] and are suggested to be involved in the pathogenesis of obesity-related VTE [8]. Leptin induces TF expression [37], while adiponectin acts in an opposite way [38]. Recently, Kimura and co-workers showed that weight gain was associated with increased leptin levels and decreased levels of adiponectin, independently of the attained BMI [39]. However, no study has yet investigated the role of adipokines in VTE.

In the present study, most of the observed VTE risk by weight gain was independent of the attained BMI, suggesting that the weight gain was more important for the VTE risk than the resulting state of obesity. Nevetheless, the negative effects of weight gain were more pronounced among subjects with BMI ≥30 kg/m^2^ indicating that the combination of obestity and further weight gain yielded a higher risk than the two components separately. According to the thrombosis potential model [40], the effect of venous stasis seen in obese individuals [9] combined with other possible procoagulant mechanisms due to adipose tissue growth may explain this phenomenon.

Weight loss and weight variability is associated with increased risk of cardiovascular disease, overall and cardiovascular mortality [12–14]. However, several reviews and meta-analyses have pointed out that the effects of intentional weight loss on these outcomes are uncertain [16, 41, 42], and confounding by concomitant illness may be of importance. The observed increase in risk of provoked VTE by weight loss in our cohort was not attenuated by exclusion of subjects with cancer, suggesting that cancer development did not contribute profoundly to the apparent association between weight loss and VTE risk. However, other chronic diseases associated with increased mortality, assessed by competing risk by death analyses, attenuated the association between weight loss and VTE risk. Thus, it is likely to assume that other concomitant illnesses inducing weight loss would affect the association.

The main strengths of our study are the prospective design, objective measurement of exposure data by trained personnel and the validation of VTE events. All hospital-care in the region is exclusively provided by a single hospital, which facilitates the completeness of our outcome registry. Some limitations should be considered. The interval between visits was six to seven years, and there would likely be some degree of exposure misclassification as subjects may have gained and lost weight between visits. Even though body weight was measured in a standardized way, variation in clothing and the time of day the examination took place might have introduced measurement bias. In addition, especially subjects with the greatest weight change could be prone to further weight change during follow-up, which could lead to an overestimation of the true associations due to differential misclassification. Further, we did not know the exact causes of weight variation, although we included models where cancer patients were censored and decreased survival was taken into account. Despite this, residual confounding could still be an issue, especially for the unexpected asssociations between weight loss and VTE risk.

In conclusion, our study suggests that weight gain, independent of attained BMI, is a risk factor for VTE. The risk of VTE associated with weight gain was particularly high in already obese individuals. Surprisingly, we also found a slightly increased risk of provoked VTE in subjects with weight loss.

## Supporting Information

S1 Table Hazard Ratios (HR) with 95% Confidence Intervals (CI) for Venous Thromboembolism (VTE) Across Quintiles (Q) of Changes in Body Weight and BMI, Stratified according to BMI (DOCX)

S1 Fig Overview of study participation and follow-up. Dots indicate participation at a given survey, arrows indicate follow-up. Subjects who attended two or more subsequent surveys were included. Subjects were followed from date of the second of two subsequent visits until the next visit, the median date of visit for the next survey (if the survey was not attended), December 31th 2012, a VTE event, migration or death whichever came first. Subjects who attended three or four subsequent surveys contributed with multiple observations.
(TIF)
