# Supplementary material for: Weight Change and Risk of Venous Thromboembolism: The Tromsø Study
Source: PLoS One. 2016 Dec 20;11(12):e0168878. doi: 10.1371/journal.pone.0168878 (PMC5173365; doi:10.1371/journal.pone.0168878)
Supplement: S1 Table — (DOCX) [file pone.0168878.s001.docx]

**Supplementary Table 1. Hazard Ratios (HR) with 95% Confidence Intervals (CI) for Venous Thromboembolism (VTE) Across Quintiles (Q) of Changes in Body Weight and BMI, Stratified according to BMI**

| **Body weight change, kg** | **Events** | **IR^*^ (95% CI)** | **Model 1^a^**  **HR (95% CI)** | **Model 2^b^**  **HR (95% CI)** | **Model 3^c^**  **HR (95% CI)** |
| --- | --- | --- | --- | --- | --- |
| *Subjects with BMI < 25 (12 497 observations)* | | | | | |
| Q1 (-37.8 to -2.2) | 33 | 2.2 (1.6-3.1) | 1.07 (0.59-1.96) | 1.15 (0.63-2.10) | 1.21 (0.65-2.25) |
| Q2 (-2.1 to 0.0) | 15 | 1.0 (0.6-1.6) | 0.71 (0.35-1.41) | 0.73 (0.36-1.47) | 0.78 (0.38-1.58) |
| Q3 (+0.1 to +2.0) | 17 | 1.1 (0.7-1.7) | Ref | Ref | Ref |
| Q4 (+2.1 to +4.5) | 13 | 0.8 (0.5-1.4) | 1.01 (0.49-2.09) | 0.99 (0.48-2.03) | 0.96 (0.45-2.04) |
| Q5 (+4.6 to +23.0) | 12 | 0.8 (0.5-1.4) | 1.45 (0.68-3.07) | 1.34 (0.63-2.87) | 1.45 (0.67-3.13) |
| *Subjects with BMI ≥ 25 (14 879 observations)* | | | | | |
| Q1 (-56.6 to -0.5) | 64 | 3.8 (3.0-4.9) | 1.66 (1.07-2.59) | 1.66 (1.07-2.60) | 1.68 (1.07-2.65) |
| Q2 (-0.5 to +2.5) | 48 | 2.6 (2.0-3.5) | 1.36 (0.86-2.16) | 1.38 (0.87-2.19) | 1.33 (0.83-2.14) |
| Q3 (+2.6 to +5.0) | 29 | 1.6 (1.1-2.2) | Ref | Ref | Ref |
| Q4 (+5.1 to +8.1) | 35 | 2.1 (1.5-2.9) | 1.66 (1.01-2.71) | 1.60 (0.97-2.62) | 1.66 (1.00-2.47) |
| Q5 (+8.2 to +40.0) | 36 | 1.9 (1.4-2.7) | 2.29 (1.40-3.76) | 2.04 (1.23-3.39) | 2.08 (1.24-3.47) |

**^*^**Crude incidence rates per 1000 person-years

^a^ Age as time-scale, adjusted for sex

^b^ Model 1 + adjusted for BMI

^c^ Model 2 + adjusted for smoking, systolic blood pressure, total cholesterol, HDL cholesterol and diabetes mellitus
