# Supplementary figures and images for: Weight Change and Risk of Venous Thromboembolism: The Tromsø Study
Source: PLoS One. 2016 Dec 20;11(12):e0168878. doi: 10.1371/journal.pone.0168878 (PMC5173365; doi:10.1371/journal.pone.0168878)

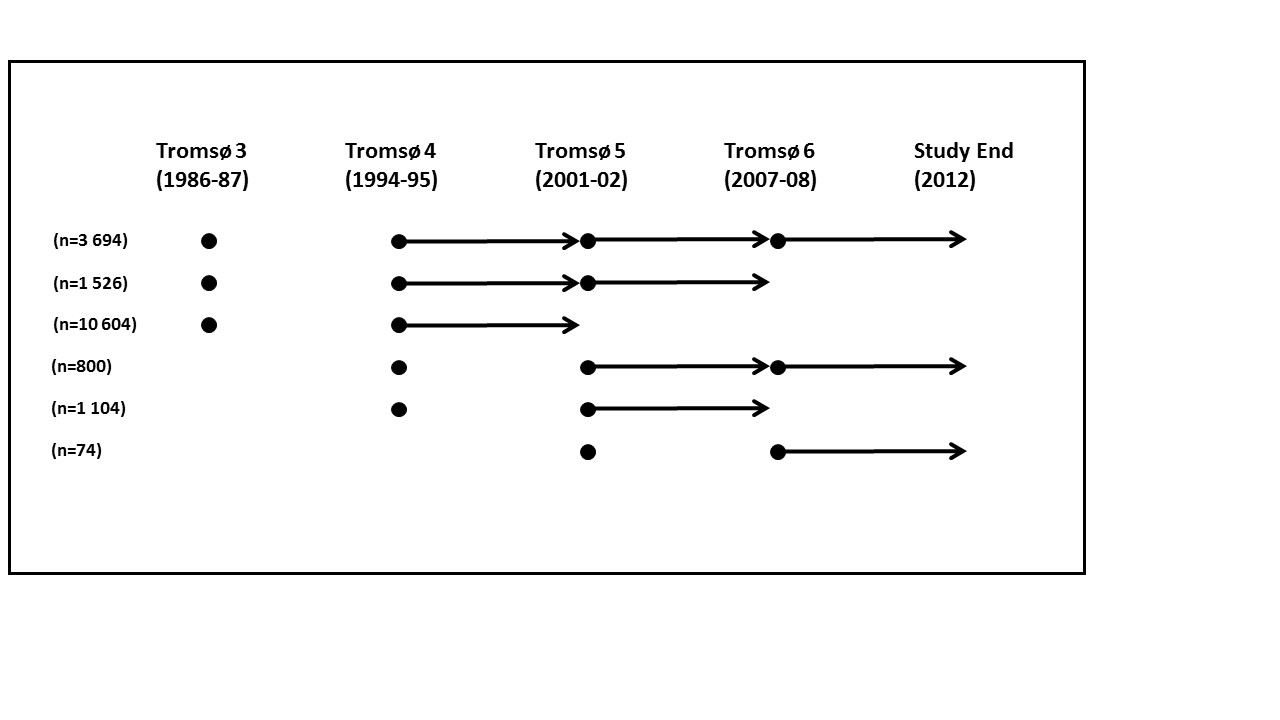

Supplement: S1 Fig — Dots indicate participation at a given survey, arrows indicate follow-up. Subjects who attended two or more subsequent surveys were included. Subjects were followed from date of the second of two subsequent visits until the next visit, the median date of visit for the next survey (if the survey was not attended), December 31th 2012, a VTE event, migration or death whichever came first. Subjects who attended three or four subsequent surveys contributed with multiple observations. (TIF) [file pone.0168878.s002.tif]
